# Supplementary figures and images for: Cortical encoding of phonetic onsets of both attended and ignored speech in hearing impaired individuals
Source: PLoS One. 2024 Nov 22;19(11):e0308554. doi: 10.1371/journal.pone.0308554 (PMC11584098; doi:10.1371/journal.pone.0308554)

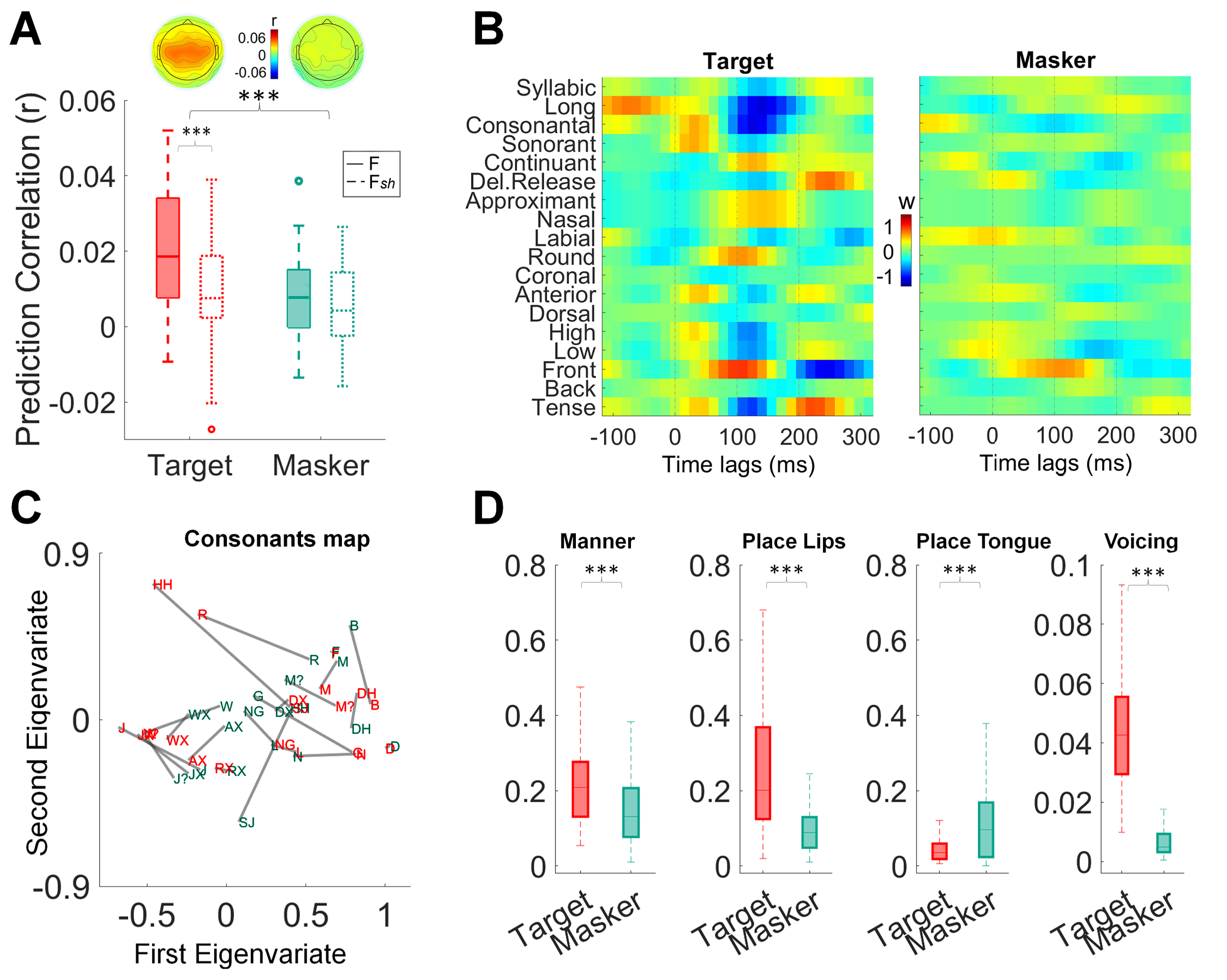

Supplement: S1 Fig — (A) EEG prediction correlations (Pearson’s r) for phonetic features models F and shuffled control Fsh of the target and masker speech. Boxplots represent the median and inter-quartile range (IQR) of the distributions. Scalp topographies represent the distribution of prediction correlations across all channels (average across participants) for the F model. Error bars indicate the SEM across participants. (B) TRF weights at channel FCz for the eighteen phonetic features for the F model. (C) Phoneme distance maps (PDMs) for target (red) and masker (green) speech. (D) EEG sensitivity to groups of phonetic features, i.e., quality of clustering of the EEG responses around relevant phonetic contrasts, for target and masker speech. (TIF) [file pone.0308554.s001.tif]

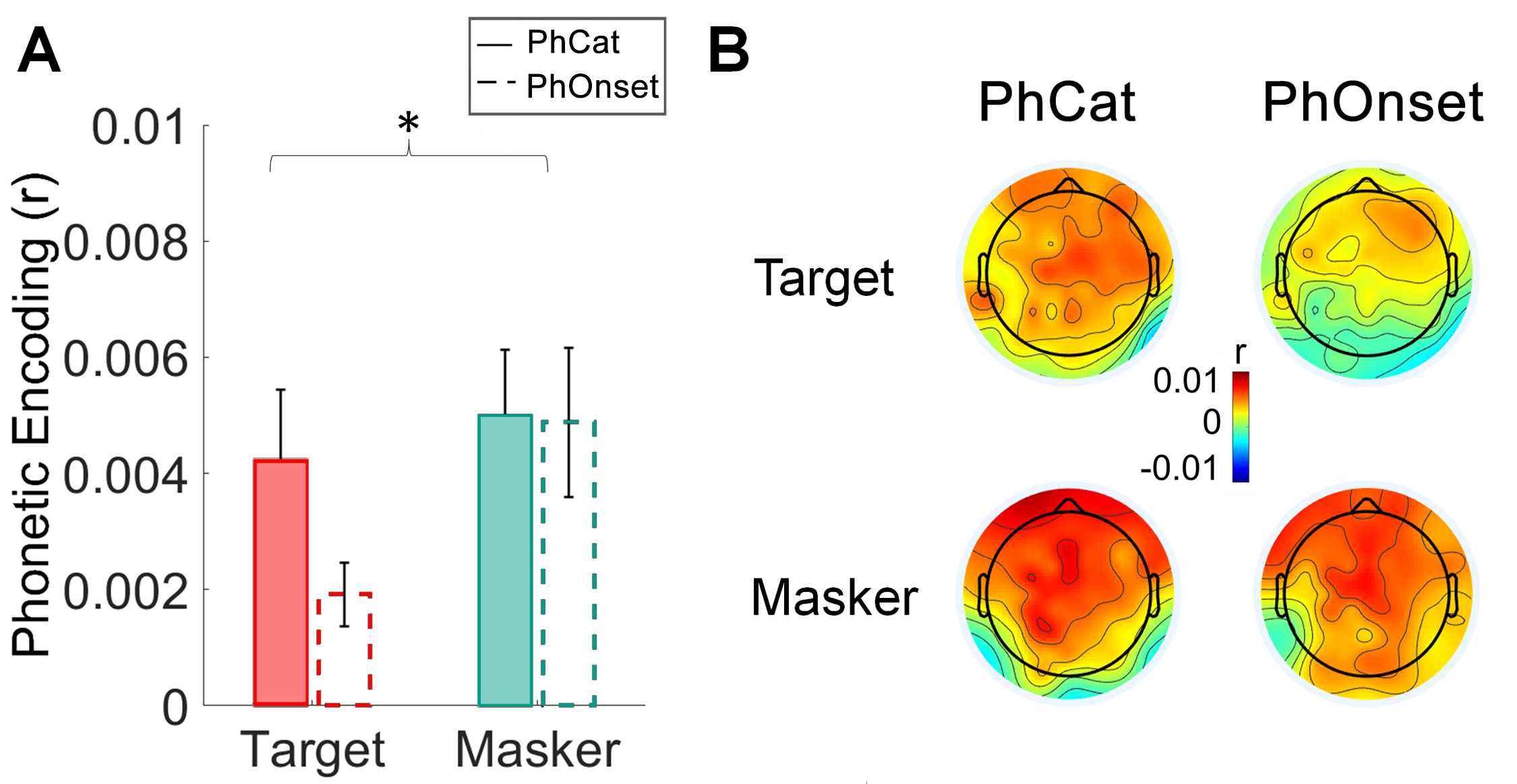

Supplement: S2 Fig — (A) EEG prediction gains obtained from the PhCat (FS-S) and PhOnset (FshS-S) metric, for the target and masker speech. Bars represent the increase in prediction correlations (r) averaged across all participants and electrodes. Error bars represent the SEM across participants. (B) Topographical distribution of the average EEG prediction correlation increases from the baseline model S, across all electrode locations. (TIF) [file pone.0308554.s002.tif]

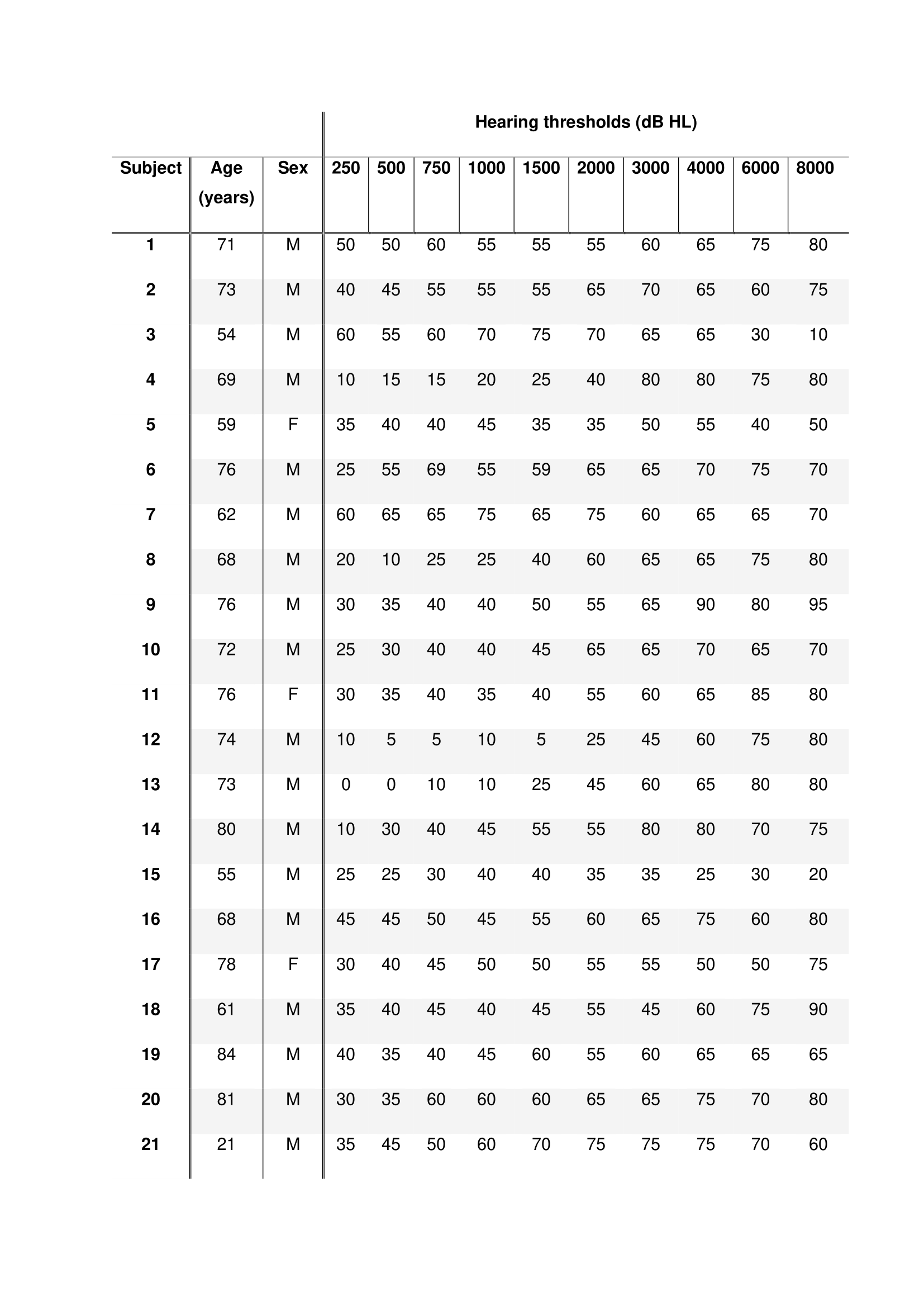

Supplement: S1 Table — (TIF) [file pone.0308554.s003.tif]
