# Supplementary material for: Cortical encoding of phonetic onsets of both attended and ignored speech in hearing impaired individuals
Source: PLoS One. 2024 Nov 22;19(11):e0308554. doi: 10.1371/journal.pone.0308554 (PMC11584098; doi:10.1371/journal.pone.0308554)
Supplement: Attachment — Submitted filename: Response to Reviewers.docx [file pone.0308554.s004.docx]

**Authors:** We thank the editor and the reviewers for their work on this manuscript. We appreciate the Editor’s observation that it is acceptable to have limitations in a study that addresses this question for the first time. We did our best to address all the concerns of the editor and the reviewers, especially by ensuring that all the constraints and limitations of this study are crystal clear, which we think will greatly benefit future work in this area. Please find our point-by-point answer below.

(Note that the line numbers refer to the document when visualising it in “simple markup” mode – as opposed to “full markup”)

**Editor Comments**

Because one of the original reviewers indicated unavailability, an additional reviewer was solicited. Now three reviews were returned, and they gave contradictory recommendations. Thus, I have examined the revised manuscript more carefully and also re-read the authors' responses to the reviews. I think I agree with all the reviewers that this manuscript adopts a novel approach (TRF) to examine cortical tracking of attended and ignored speech in individuals with hearing impairment and has the potential to make a significant contribution to the field. Being a first attempt in this direction, this study also has limitations (e.g., large range of age differences, lack of a control group) that should be properly addressed and acknowledged. Thus, I'd like to recommend the following action from the authors, apart from addressing the specific comments from the reviewers:

1. **E.** I checked Aličković et al. (2021) and couldn't find a detailed report of the participant information. Thus, I'd suggest the authors add a table in the current manuscript (or in supplementary materials), detailing the age, gender, and hearing loss level for each of the 34 participants.
A. We added a table as Supplementary Information of the Participants section with a detailed overview of participant information.
2. **E.** Age difference is indeed a concern, as hearing and auditory processing systems change dramatically over age. As the participants aged between 21 and 84 years, it is questionable whether the findings of the current study are primarily driven by a particular age group (e.g., old adults), or whether it apply universally to all ages. If the authors have had more participants, they could try to divide the participants into age groups, but that's not realistic here. I suggest the authors include age as a covariate into the statistical analyses (e.g., three-way ANOVAs) and check if age dramatically changes the results. Alternatively, if the participants are mainly older adults, perhaps the authors could conduct analysis on a subset of the data by excluding young adults, but the sample size will become smaller. Please also comment on how age difference may have impacted the results and the interpretation, as a limitation (apart from the sentence on line 500-502).
A. We agree that the age range in this experiment appears to be quite wide, going from 21 to 84 years. One issue that we clarified in the text is that the dataset included data from a 21-year-old male and a 45-year-old female, which widen the range greatly. Excluding those participants, the age range is a much more reasonable 54-84. The results did not change after excluding the two younger participants from the dataset. Please find below the details of this re-analysis (in brief, the statistical results were unaffected).

Re-analysis results

As previously done, we employed a repeated three-measures ANOVA to understand the impact of three factors: Noise Reduction (Off or On), Attention (Masker and Target), and Model (F vs. Fsh) or Model Gain (PhCat vs. PhOnset).

For phonological encoding, our analysis revealed a significant effect of attention (*F*(1,31) = 23.27, *p* = 3.82e-05, ηp² = 0.44), with larger EEG prediction correlations for the target compared to the masker speech (r_target_ > r_masker_, post-hoc *t*-test: *t*(31) = 4.82, *p* = 3.82e-05, Cohen’s *d* = 0.73). There was also a main effect of model (*F*(1,31) = 34.39; *p* = 2.04e-06, ηp² = 0.53), with the F model showing greater EEG prediction correlations than its control model, F*sh*_,_ derived by re-running the TRF computation after shuffling the corresponding phoneme categories (r_F_ > r_Fsh_; post-hoc *t*-test: *t*(31) = 5.86, *p* = 2.04e-06, Cohen’s *d* = 0.61); and a significant Attention*Model interaction (*F*(1,31) = 18.23, *p* = 1.81e-04, ηp² = 0.38). No significant effect of Noise Reduction emerged from the ANOVA.

For phonetic category and phoneme onset encoding, our three-way repeated-measures ANOVA revealed no significant difference between the two model gains (PhCat and PhOnset metrics) (*F(*1,31) = 3.47, *p* = 0.07, ηp² = 0.1). A significant effect of Attention also emerged (*F(*1,31) = 9.4, *p* = 0.005, ηp² = 0.24), with greater EEG prediction gains in the masker compared to the target (r_masker_ > r_target_, post-hoc *t*-test: *t*(31) = 3.07, *p* = 0.005, Cohen’s *d* = 0.39). Once again, there was no main effect of Noise Reduction. An interaction between attention and model gain did reach statistical significance (*F(*1,31) = 4.81, *p* = 0.037, ηp² = 0.14).

3. **E.** I think it'll be a good idea to acknowledge the lack of control group as another limitation of the current study.
A. We agree and have strongly highlighted that limitation at the end of the Discussion section (lines 578-580).
4. **E.** In addition, I have a comment about the use of phonological processing and phonetic features.

   In the abstract, the authors proposed a hypothesis of phonological encoding difficulty. However, this hypothesis was not explained further in the introduction, especially what measures would indicate phonological and non-phonological encoding, and the specific predictions about these measures. Also, the authors did not return to this hypothesis in the discussion, to discuss whether the results confirmed this hypothesis or not, and if so, whether fully or partly, etc. Throughout a large part of the manuscript, the authors used a different term "phonetic features" and seemed to use it against acoustics. I wonder what's the relationship between "phonetic features" and phonological encoding. It is true that phonology and phonetics are sometimes used interchangeably in the literature, but to many people they're distinct (e.g., phonology referring to abstract phonemes, whereas phonetics includes variations across speakers, phonetic contexts, etc.). It will be helpful if the authors could elaborate on the relationship between these terminologies if they're intended to be different, or, if they were intended to mean the same thing, then use one terminology consistently across the manuscript. I also have some issue with the conclusion that the results confirmed the hypothesis (again this was only briefly said in the abstract and not carefully discussed in the discussion). To many people, phonological encoding means abstract, acoustically invariant phonemes. However, following this definition, the results did not provide evidence for the encoding of acoustically invariant features in the target and masker speech streams. What the results showed was something very specific, the encoding of phoneme onsets. Thus, I don't think it's appropriate to simply conclude that the results confirmed the hypothesis.
A. This is a very important point. Therefore, we have revised our use of the terms “phonological encoding”, “phonetic features” and “phoneme onsets” throughout the manuscript, to ensure that they are used consistently and in a way that clearly reflects our intention.

"Phonetic features" are used when we refer to the methodology, as we used them as features for the TRF model. By “phoneme onsets” we intend phonetic boundaries, which in practice consist of timestamps marking the start of a phonemic unit. By the neural encoding of “phonological” information we refer to the encoding of information that relates to phonemes, from phoneme onsets to acoustically-invariant responses to categories.

**Reviewer #1**

Thank you for taking the time to thoroughly read the manuscript.
As was already said, the topic is significant, the article is well-written, however there are still unanswered questions. Although there was a legitimate worry about the way the hidden material was presented, the study cannot be published in its current form due to significant methodological issues.

1. R. The wide age span that includes everything from young adults to the elderly. This is a crucial factor because it is well known that the ability to perceive speech declines with age, which is why the volunteers were not well chosen.

**A.** We agree with the Reviewer on the importance of the appropriate choice of participants depending on their age range. We verified that this is not a problem in our study. Only two participants out of thirty-four were below fifty years of age (one 21y.o. male and one 45y.o. female). Removing them from the dataset leads to a more consistent age range (54-84), without changing the result (please refer to the reply to the editor for further details on that re-analysis).

2. R. Another significant factor influencing how speech sounds are perceived is the difference between the sexes. A quick report on the information from the hearing assessments is given. This data is essential to comprehending the characteristics of the participants and the rationale for the conclusions.
A. We have provided a detailed description of participant’s sex, age, and hearing profile, inserted as a Supplementary Table of the Participants section.

Without concentrating on participant data, the article's emphasis is in providing a detailed presentation of the technology. Nonetheless, the article entails assessing and analysing the responses provided by the participants. which the writers mostly disregarded.

I heartily advise that a fresh investigation be conducted, but this time it should consider the research participants.

**A:** We share the Editor’s view on this point. We respectfully disagree with the reviewer, as we think that the article has merit despite its limitations. In fact, in addition to proposing a provocative view on how the neural processing of speech might work in HI individuals, our findings provide valuable insights that will be precious for future investigations. Considering the difficulty with the acquisition of this kind of data on this population, we think this makes our study valuable in itself. Indeed, we have done our best to improve the manuscript so that it clearly indicates its limitations and recommendations for future work in this area.

**Reviewer #3**

This manuscript addresses a significant topic, employing temporal response function (TRF) analysis to explore cortical responses to phonological features within sentences among individuals with hearing impairments. It offers novel insights into the auditory attentional selection mechanisms related to hearing impairments.

1. R. The description of the results in the Abstract is somewhat unclear: “Multivariate temporal response function analyses indicated a stronger phonetic-feature encoding for target than masker speech streams. Interestingly, robust EEG encoding of phoneme onsets emerged for both target and masker streams, in contrast with previously published findings with normal hearing participants (NH) and in line with our hypothesis that speech comprehension difficulties emerge due to a robust phonological encoding of both target and masker. Finally, the neural encoding on phoneme-onsets encoding was stronger for the masker speech, pointing to a possible neural basis for the higher distractibility experienced by individuals with HI.”
   These sentences introduce the results from three minor perspectives, but it's unclear whether phonetic features, phonological features, and phoneme onsets are independent. At least, I believe that phoneme onsets are a type of phonological feature, so the Abstract's depiction of the results seems confusing. In addition, it would be better to add some logical expressions instead of merely using terms like "Interestingly" and "Finally" to relate the results.

**A.** We agree that utilising a clear and consistent terminology is very important. We revised that issue throughout the paper. Regarding the Abstract, we restructured it to better reflect our hypothesis, ensuring that it is consistent with the terminology used in the manuscript.

2. R. Organizing the Introduction into paragraphs would make it clearer and more logically structured for the reader.

**A.** We organised the Introduction into paragraphs with a clear narrative.

3. R. “All participants were native Danish speakers and had mild-to-moderately severe symmetrical sensorineural hearing impairment, with an average 4-frequency Pure Tone Average threshold of 47.5 dB hearing level.”
   It would be beneficial to provide information on the degree of hearing loss to see if there are differences among individuals with varying levels of hearing impairment. At least, authors should provide the standard deviation of 4-frequency Pure Tone Average threshold of 47.5 dB hearing level.

**A.** We agree with the Reviewer that providing the specific hearing thresholds for each participant would offer a more complete picture of their hearing profile. Therefore, we have added a table as Supplementary Information to the Participants section where we report single-subject hearing profiles, together with age and sex information.

4. R. Why was only 10% of the corpus manually checked? What was the focus of this manual check, and how can you ensure the remaining 90% of the corpus is problem-free and meets the requirements?
A. In order to check the accuracy of phonetic alignment, it is usually sufficient to check the initial, middle, and final section of each audio file, in search for mismatches between the identified phonemes and the actual sounds present in the audio at that time window. If the procedure appears to be correct in these three main sections, it is likely that the intermediate portions of the audio file were also well aligned, since any problems would have resulted in a temporal shift of the detected phoneme time stamps, with alignment errors carrying over to the end of the file. So, the 10% provides a good estimate of the general performance of the forced aligner. Please note that these aligners are very precise on speech material like the ones used in this study. They can be problematic in more dynamic scenarios involving turn-taking and filler sounds (e.g., uhm), which was not our case.
5. R. How were the features shuffled (from F to Fsh), and how were the phoneme onsets extracted? Please add detailed information about signal processing.
A. Phonetic feature shuffling consisted in randomly shuffling the phonetic feature category, while keeping the time stamps constant. Such a model, where the temporal information is retained but the phonetic categorical identity is disrupted, only carries phoneme onset information, since the time of occurrence of phonemes is the only information that is left intact, but it has the same dimensionality of the unshuffled model. This methodology has been often adopted by our team and other labs in numerous previous studies (e.g., Edmund Lalor’s team, Jonathan Simon’s team, Nima Mesgarani’s).

We have clarified the shuffling procedures and the relation to the model names throughout the *Speech feature models* section of the manuscript.

6. R. What does PhOnset mean, and what is the definition of FshS?

**A.** We have clarified that in the text, since we agree that it could be confusing. PhOnset does not refer to a particular model of feature set, but to a contrast between two models (FshS - S).

FshS consists of temporally shuffled phonetic features (Fsh) and Spectrogram (S). The difference between the FshS model’s prediction correlations and those obtained from a model consisting of Spectrogram only, will represent the unique contribution of phoneme onsets. This prediction correlation gain, FshS - S, is called the PhOnset metric, because it quantifies the prediction gain due to phoneme onsets when accounting for the acoustics.

7. R. Figure 2c's resolution is not high enough, making the image somewhat blurry. Could you explain in detail the value of the phonetic distance feature in the article? Should the article also discuss the brain processing mechanisms of phonetic distances? Additionally, why are the points in the consonant map inconsistent between Fig 2c. and Fig S2c? What does the "?" in the consonant map represent?
A. The consonant maps in Figs 2C and S2C represent the relative distance of brain responses in response to different phonemes. This concept is based on the idea that neural responses should be more similar for similar sounds, hence the possibility of plotting a map where sounds represented with similar brain activity are plotted closer to each other. Therefore, in terms of neural processing, the only information that the consonant maps are representing is the relative distance between the brain encoding patterns of these sounds. The way the maps are built is by applying multi-dimensional scaling on the TRF weights, projecting each set of nFeature-by-nSamples-by-nElectrodes onto a lower-dimensional space. This dimensionality reduction is performed on TRF weights which were fit separately for Target NR Off, Masker NR Off, Target NR On and Masker NR On conditions. Then, for NR Off and NR On separately, we considered the Target distance map as a reference, and we mapped the corresponding Masker consonant map to it, with isomorphic scaling. For each of NR Off and NR On, this allowed us to visualise Target and Masker maps on the same scale, but since the rescaling is performed on the two noise reduction conditions separately, the NR Off map in Fig 2C and the NR On map in S2C are not directly comparable, because they were projected onto different spaces. This is why the points where the consonants are located look inconsistent between the two maps.

Figure 2C’s resolution has been improved and the colour contrasts made more intuitive.

The”?” symbol that follows some consonants is a rendering of the corresponding IPA symbol indicating the Danish phonation feature “Stød”. This kind of phonation is a type of glottal stop, related to stress and prosody. We have clarified in the text that the symbols refer to IPA (lines 334-336).

8. R. The author only included the results of the NR ON condition in the supporting information and did not calculate or discuss in detail the differences in the brain's decoding schemes between NR ON and OFF conditions. I believe this discussion is meaningful and necessary. Why not discuss the TRF encoding response under the conditions of the noise reduction algorithm being turned on and off in more detail?

**A.** The use of a different noise reduction scheme impacted the behavioural performance of participants, who scored significantly better on 2-choice questions when the noise reduction algorithm was turned on (lines 346-348). At the neural level, no significant difference between NR Off and NR On emerged. For that reason, we found that focussing on NR Off in the main text made the manuscript easier to read, but we do report NR On results as supplementary figures.

We could speculate that the fact that no effects of amplification scheme emerged could indicate that our result reflects the general processing strategy for phonological information in HI participants, rather than something that could be affected in the short term by a particular amplification scheme. However, further studies are necessary to support that claim, so we decided to report this data for now, without overselling a null result.

9. R. Furthermore, the author should provide behavioural results (such as the correct rate of multiple-choice questions in the experiment) to prove that the participants were conducting the experiment according to the instructions. If the experimental subjects were focused on a mixed speech stream or the wrong speech stream, it does not exclude the possibility of obtaining the results reported in this paper (both target and ignore speech streams have significant cortical tracking effects, and the phoneme-onset response to the ignore speech stream is stronger). Therefore, the experimental conclusions of this paper have not been fully substantiated. The author could also provide more EEG analysis results to prove that the participants indeed focused on the correct speech stream as per the experimental setup.

**A.** This is a very important point for any study on cocktail-party speech listening. Even when instructions are clear and participants are expected to focus on one particular speech stream, it is well possible that the attention is not maintained for the whole duration of the trial, or that there are moments of distractions. A recent study in ECoG (Raghavan et al. (2023). Distinct neural encoding of glimpsed and masked speech in multitalker situations. *PLoS biology*) found that even Masker’s phonetic features are neurally encoded when the Target is silent enough to allow to glimpse the Masker out of the auditory mixture. This suggests that sustained attention on the Target is less stable and rigid than what is usually assumed in the literature. However, it is still possible to infer the overall attentional focus of participants, for instance by looking at behavioural performance, or by looking at the overall neural encoding of the Target compared to the Masker. In our study, participants seemed to be following the expected speech stream, as they were able to correctly reply to questions regarding the Target speech 73% and 84% of the time, for NR Off and NR On conditions respectively. Moreover, our TRF analysis shows that the EEG can be significantly better predicted with a Target model, compared to a Masker model, showing the expected attentional bias. This does not exclude that the attentional behaviour might have been more erratic than what can be quantified with our analyses. As such, this factor might part of the interference we refer to and help explain the robust encoding of the Masker’s phoneme onsets, as the Reviewer rightly suggests. We have added considerations on this topic at lines 541-549.
